# Supplementary material for: Structural insights into Cir-mediated killing by the antimicrobial protein Microcin V
Source: Commun Biol. 2025 Oct 9;8:1449. doi: 10.1038/s42003-025-08846-7 (PMC12511343; doi:10.1038/s42003-025-08846-7)
Supplement: Supplementary file 1 — Supplementary Information [file 42003_2025_8846_MOESM1_ESM.pdf]

Supplementary information for

Structural Insights into Cir-mediated Killing by the Antimicrobial Protein Microcin V

Stavros A. Maurakis[1], Angela C. O'Donnell[2], Istvan Botos[1], Rodolfo Ghirlando[1], Bryan W. Davies[2]*, Susan K. Buchanan[1]*

[1]Laboratory of Molecular Biology, National Institute of Diabetes and Digestive and Kidney Diseases, National Institutes of Health, Bethesda, MD, USA

[2]University of Texas at Austin, Department of Molecular Biosciences, Austin, TX, USA

*Email: susan.buchanan2@nih.gov
bwdavies@austin.utexas.edu

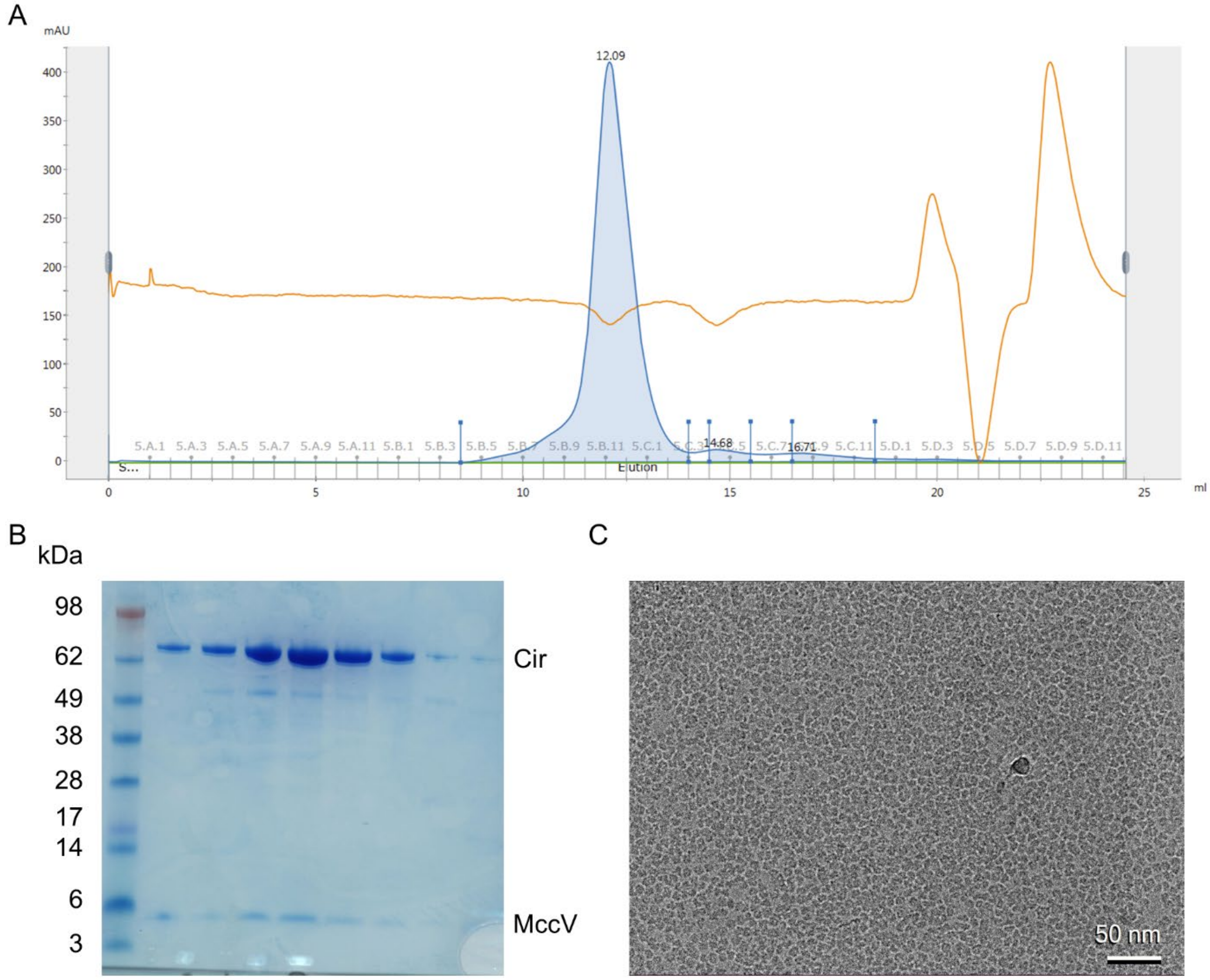

Supplementary Figure 1 – Sample Purity of Cir/MccV Complex. A) Size exclusion chromatography elution profile for Cir/MccV complex showing protein eluting as a clean, single peak. B) Coomassie stained protein gel showing relevant fractions from panel A. Fractions contained both Cir and MccV. C) Representative micrograph from cryo-EM data collection showing particle density and distribution in thin ice. Sample was loaded at 7.8 mg/mL.

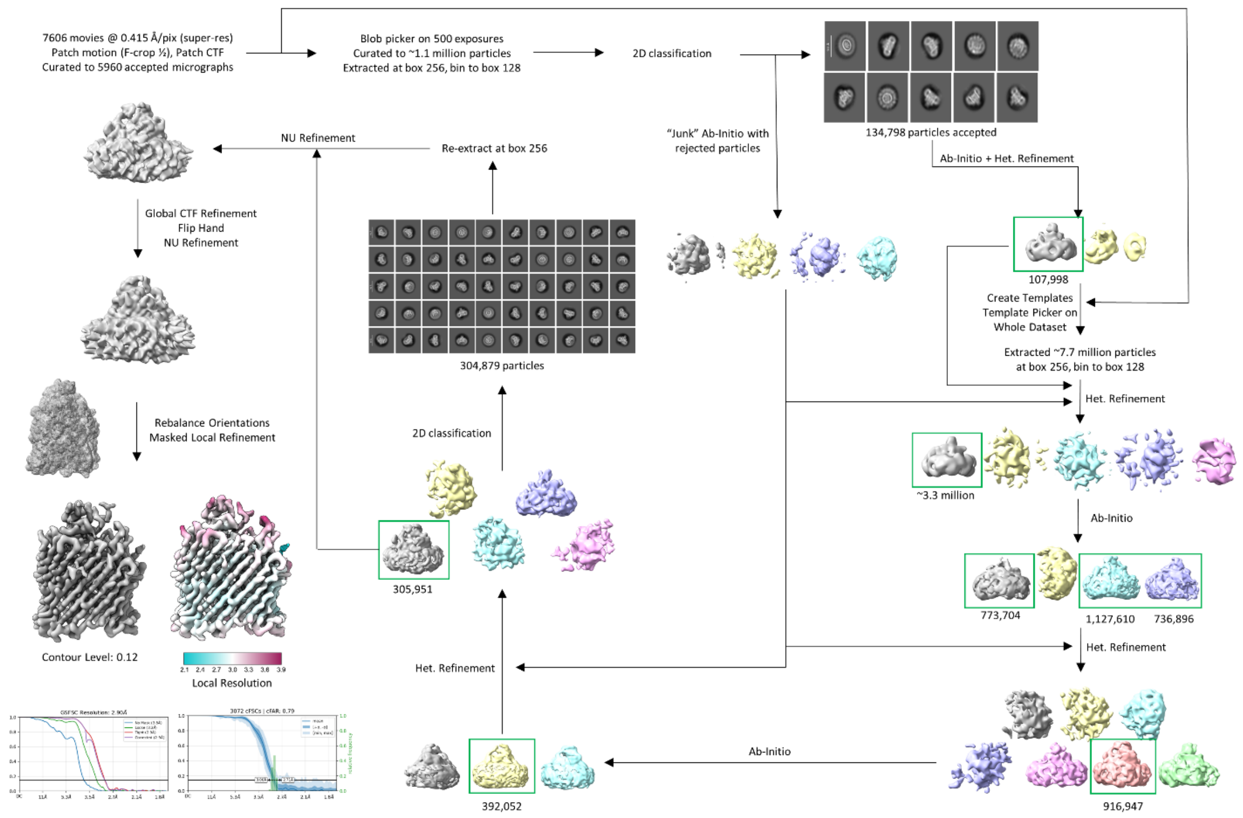

Supplementary Figure 2 – Schematic diagram of cryo-EM image reconstruction workflow for Cir/MccV RBD complex, resulting in a 2.9 Å consensus map.

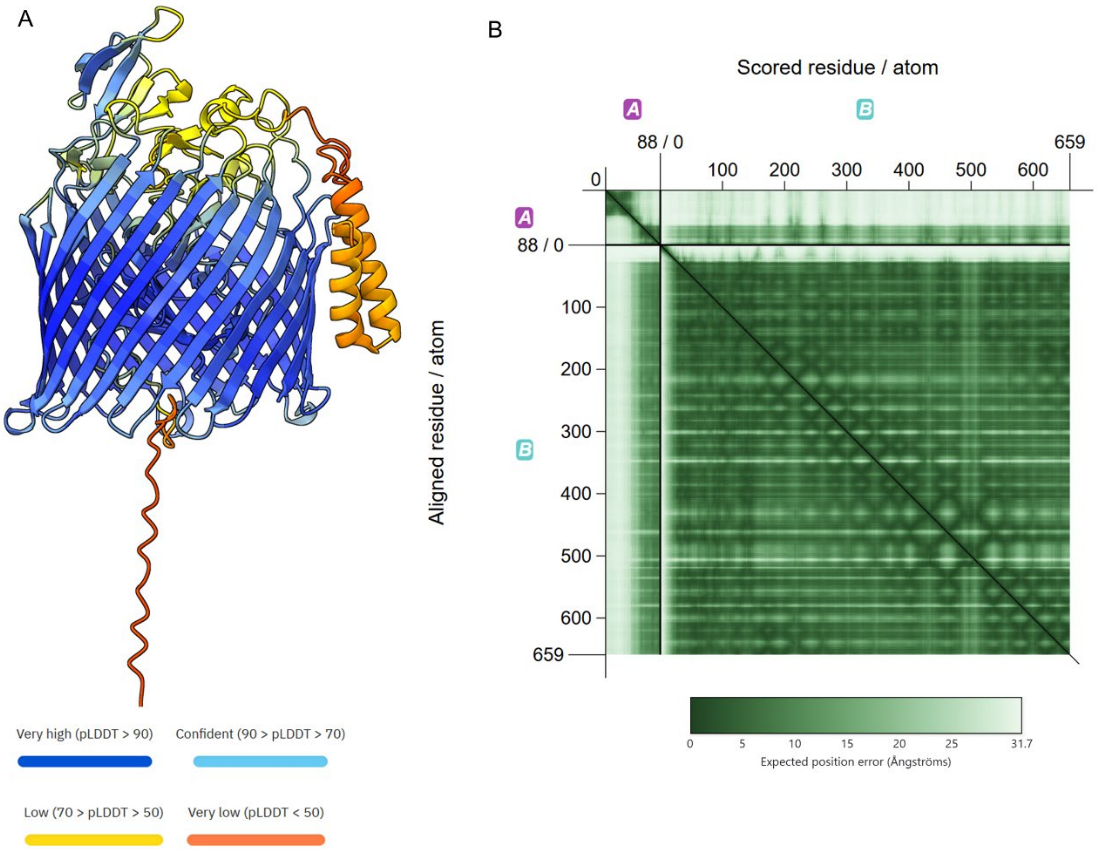

Supplementary Figure 3 – AlphaFold 2 Prediction of Cir/MccV complex. A) The predicted model for the Cir/MccV complex generated by AlphaFold 2 and colored according to pLDDT. B) PAE matrix for the model shown in panel A, showing alignment scores and expected position error per residue.

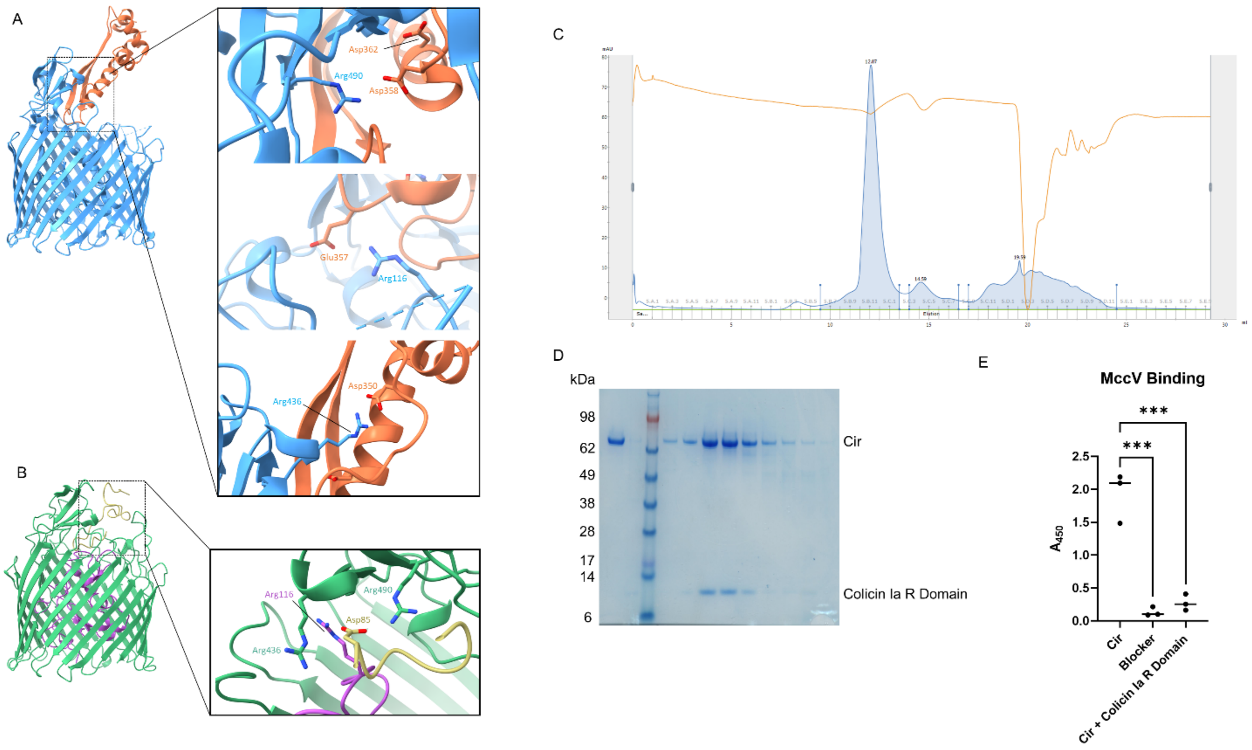

Supplementary Figure 4 – Comparison of MccV and Colicin Ia Interactions With Cir. A) Structure of Cir liganded by the receptor binding domain of Colicin Ia (PDB code 2HDI). Zoomed box shows the three positively charged arginine residues corresponding to those described for the Cir/MccV structure in figure 4. B) Cir/MccV structure and zoom of charged residues for comparison. C) Size exclusion chromatography trace from co-purification of Cir and Colicin Ia R Domain. The complex eluted as a sharp peak at ~12 mL with a broader later peak assumed to be unbound R Domain. D) Coomassie stained protein gel showing co-elution of Cir and Colicin Ia R Domain from relevant fractions shown in panel C. E) Competitive Cir binding by MccV and Colicin Ia R Domain. 1 µM of His-tagged Cir (apo and in complex with Colicin Ia R Domain) was seeded onto nickel-coated ELISA plates and allowed to bind. Following seeding, wells were blocked with 5% BSA and then probed with 1 µM biotinylated MccV. Wells were washed and then further probed with HRP-conjugated streptavidin. Following another wash, HRP signal was developed by addition of TMB substrate and coloration proceeded for three minutes. The reaction was stopped by addition of 0.5 N HCl and then absorbance at 450 nm was recorded. Statistical significance was determined for the triplicate experiments via one-way ANOVA (***, $p<0.0005$).

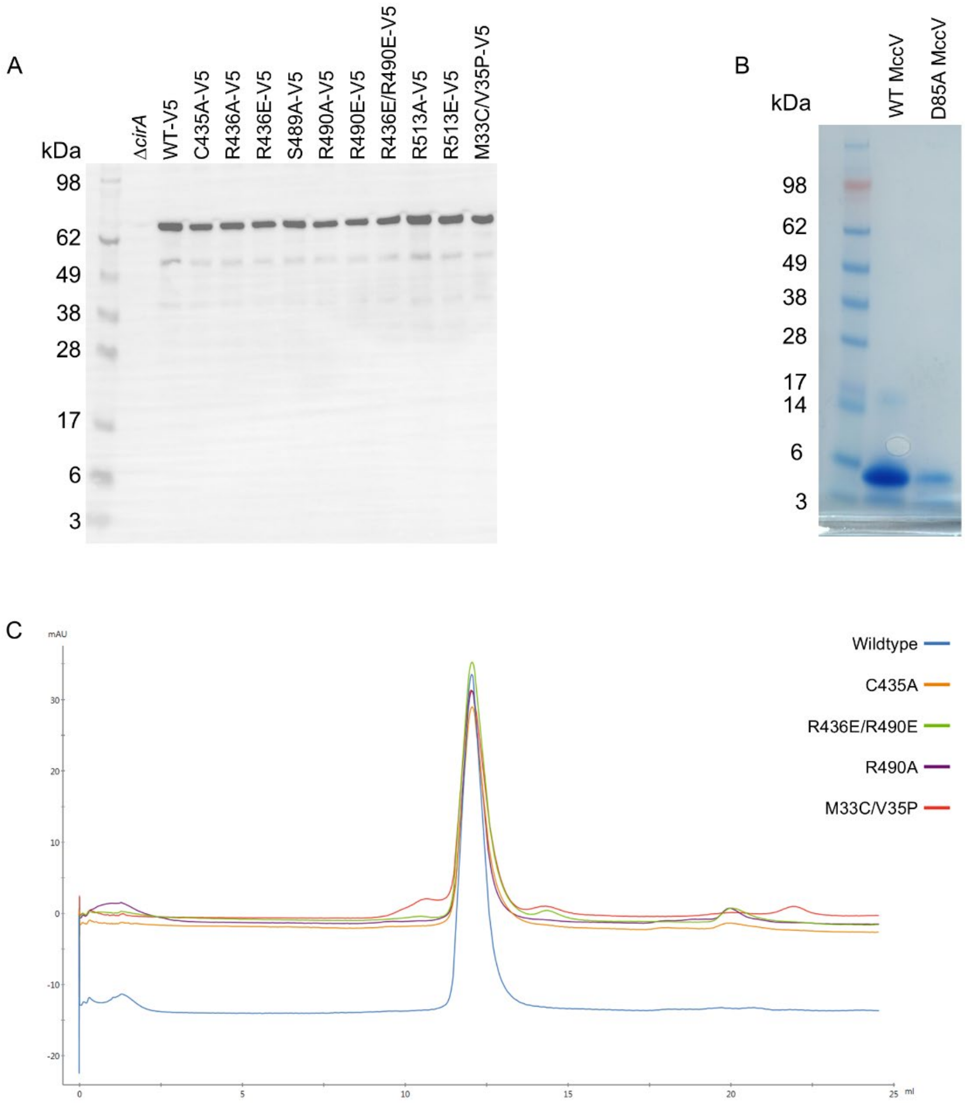

Supplementary Figure 5 – Cir Mutants Are Stably Expressed and Purified. A) Western blot showing comparable levels of Cir mutant expression and lack of detectable degradation. Proteins were detected using C-terminal V5 tags. B) Coomassie stained protein gels of WT, D85A, and D85R MccV. C) Overlay of size exclusion chromatography elution traces for WT, C435A, R490A, R436E/R490E, and M33C/V35P Cir. Proteins all eluted at the same retention volume and as clean, single peaks.

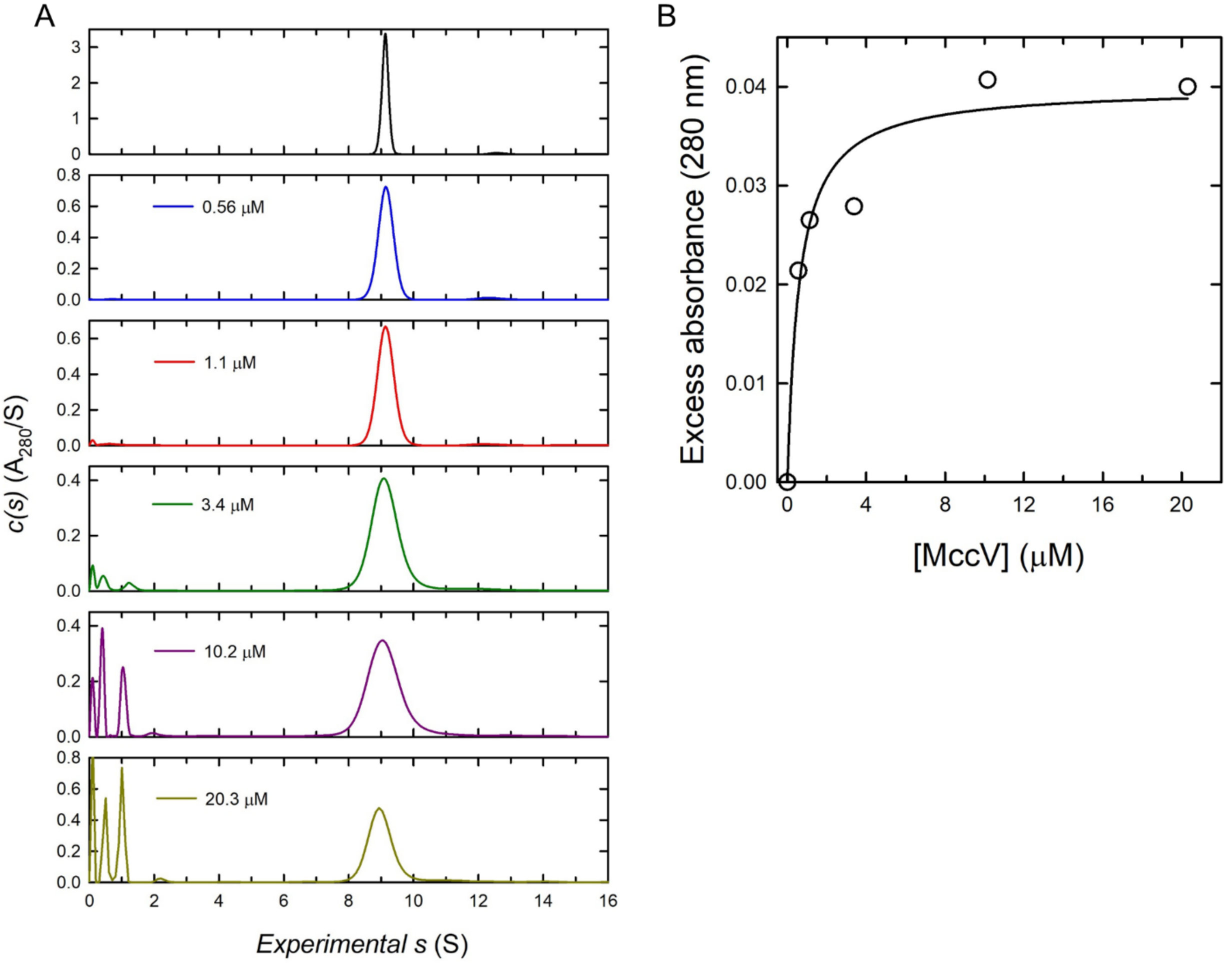

Supplementary Figure 6 – Analytical Ultracentrifugation Analysis of MccV Binding to Cir. A) Absorbance sedimentation c(*s*) profiles for Cir. Top panel - Absorbance sedimentation c(*s*) profile for NAPol solubilized Cir at 5.6 μM showing a species at 9.11 S. A membrane protein analysis returns a protein mass of 75 ± 13 kDa, supporting a Cir monomer, and a complex mass of 195 ± 41 kDa. The excess mass represents the NAPol contribution. Lower Panels - Absorbance sedimentation c(*s*) profiles for NAPol solubilized Cir at 2.7 μM in the presence of MccV. The concentration of MccV added is indicated on each plot. The absorbance signal for the Cir species provides a measure of MccV binding, which was obtained by integration. B) Binding of MccV to Cir. Excess absorbance of the Cir c(*s*) absorbance contribution, indicating MccV binding, is plotted as a function of the MccV concentration. Data were fit to a single binding model to obtain a dissociation constant KD of 0.6 ± 0.2 μM. The maximum excess absorbance at high MccV concentrations corresponds to the value expected when a single MccV binds to 2.7 μM of Cir, validating the 1:1 binding.

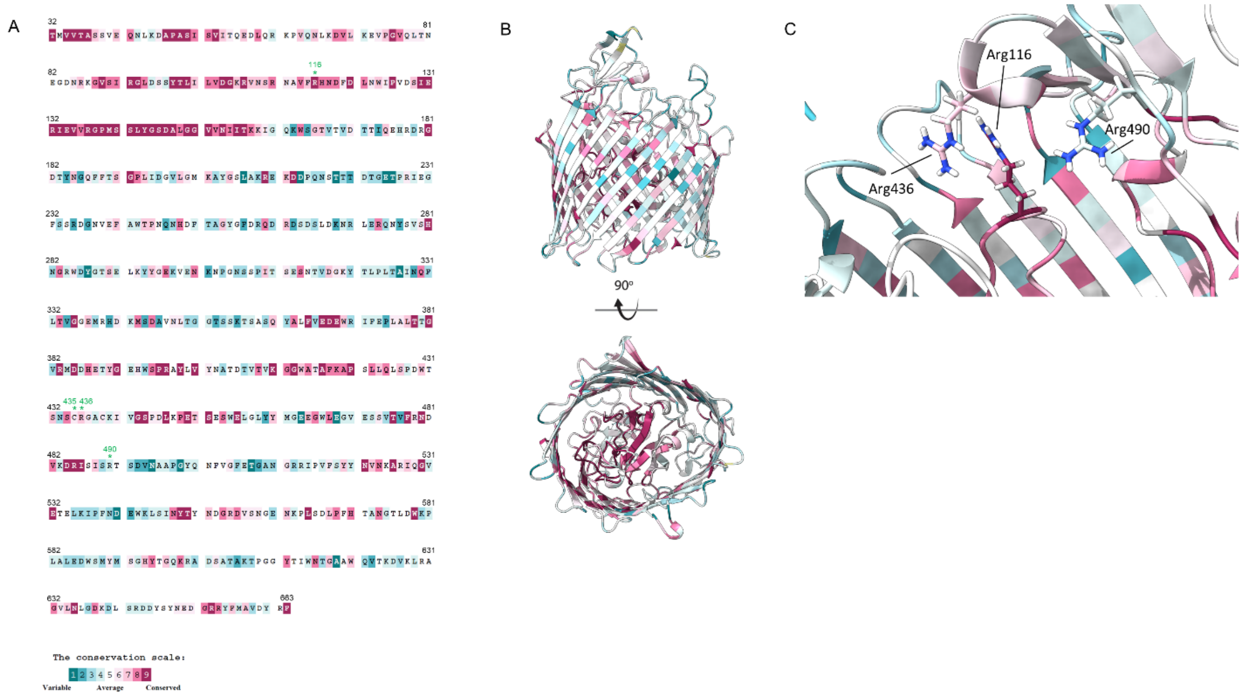

Supplementary Figure 7 – Amino Acid Sequence Conservation of Cir. A) The Cir amino acid sequence (beginning at mature start) was submitted to the ConSURF server for prediction of conserved functional regions, with a focus on Arg 116, Cys 435, Arg 436, and Arg 490. The sequence is colored according to the conservation scale shown at the bottom, and the relevant residues are highlighted with a green asterisk. B) Cir/MccV model colored according to conservation data in A. C) Conservation of R116, R436, and R490.

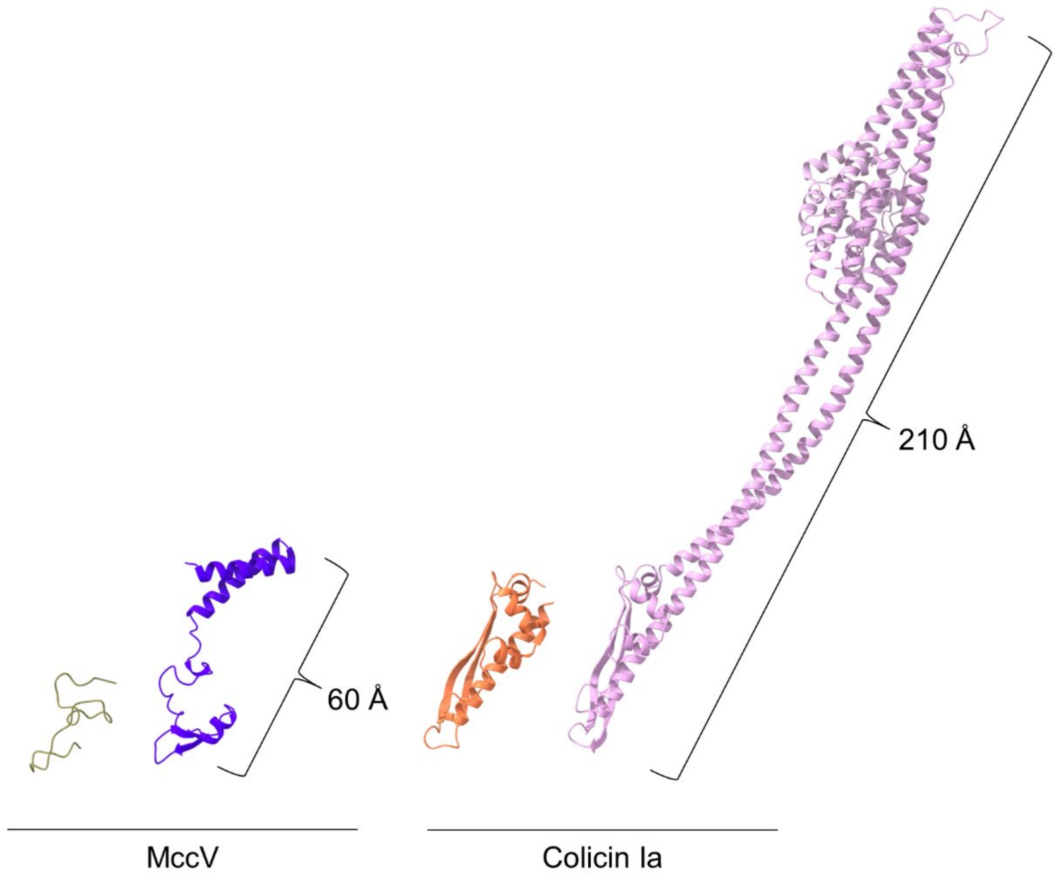

Supplementary Figure 8 – Size Comparison of MccV and Colicin Ia. Structures of (left to right) the MccV RBD, mature MccV (AlphaFold2 prediction), Colicin Ia RBD (from PDB 2HDI), and whole Colicin Ia (PDB 1CII).

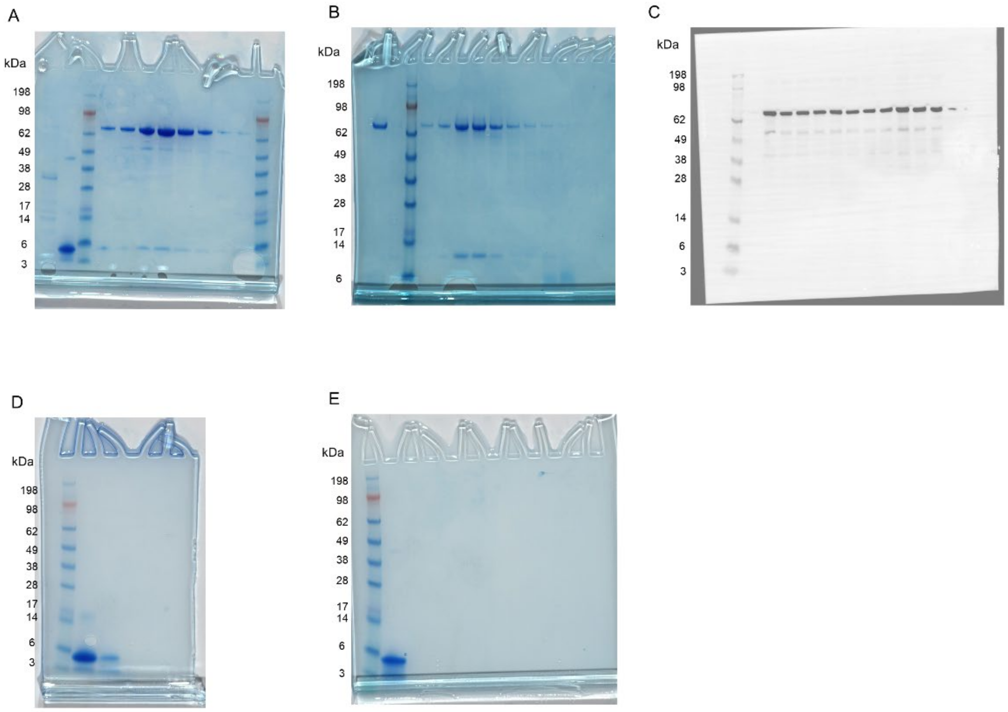

Supplementary Figure 9 – Uncropped Gels and Blot. A) Cir/MccV co-elution, cropped version shown in Supplementary Figure 1. B) Cir/Colicin Ia R Domain co-elution, cropped version shown in Supplementary Figure 4. C) V5-tagged Cir mutants western blot, cropped version shown in Supplementary Figure 5. D) WT (left) and D85A (right) MccV, cropped version shown in Supplementary Figure 5. E) D85R MccV, cropped version shown in Supplementary Figure 5.

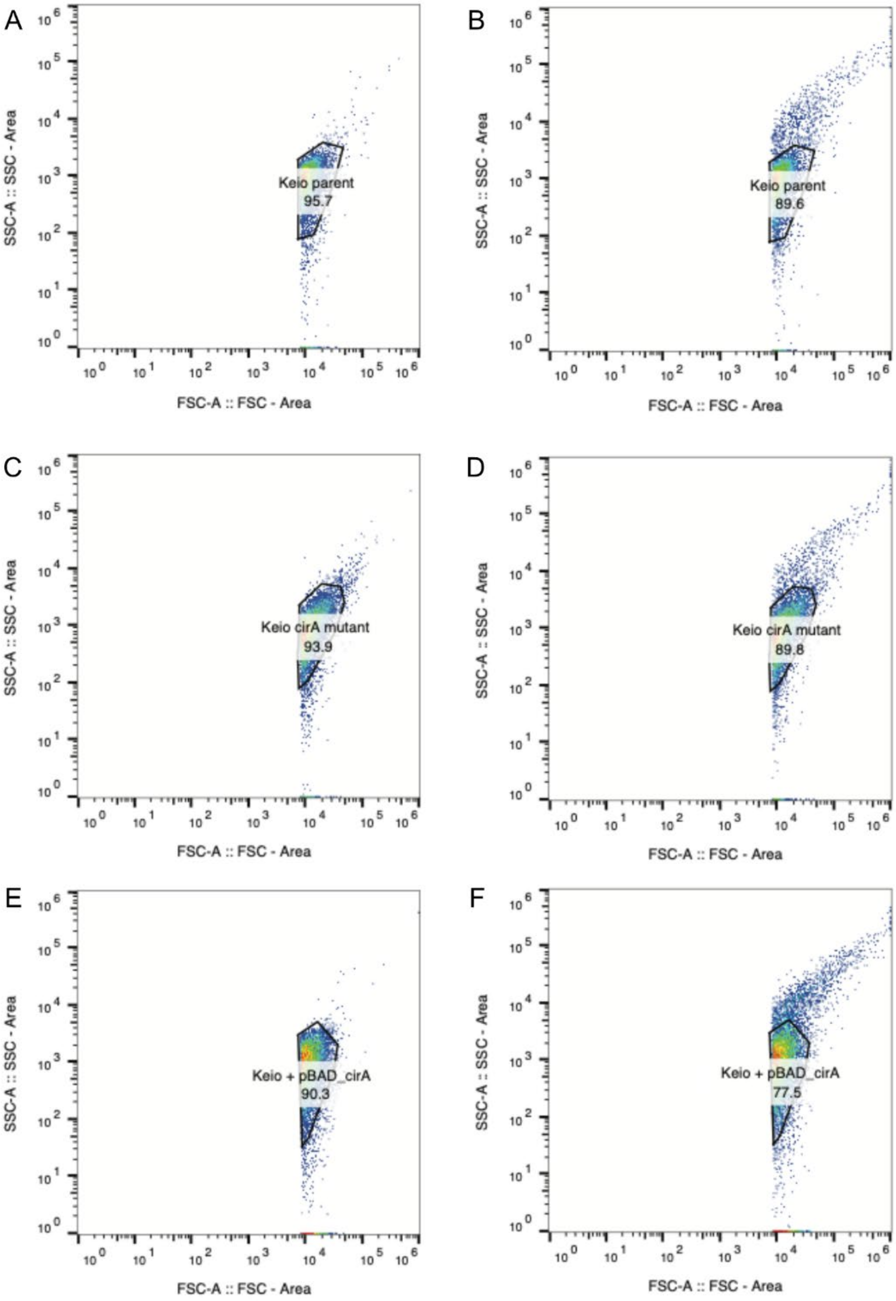

Supplementary Figure 10 - Gating strategies for flow cytometry. Scatterplots show forward scatter area (FSC) and side scatter area (SSC), which were used to identify main cell populations and eliminate debris from downstream analyses. A) Scatterplot of Keio parent cells. B) Scatterplot of Keio parent cells treated with dansyl-MccV RBD. Gating from A was applied to this population. C) Scatterplot of Keio Cir mutant cells. D) Scatterplot of Keio Cir mutant cells treated with dansyl-MccV RBD. Gating from C is overlaid on these data. E) Scatterplot of Keio strain overexpressing *cirA* from a plasmid. F) Scatterplot of Keio strain overexpressing *cirA* and treated with dansyl-MccV RBD. Gating from E was applied to these data. In each graph, the percent of data retained after gating is shown below the gating name.
