# Supplementary material for: Structural insights into Cir-mediated killing by the antimicrobial protein Microcin V
Source: Commun Biol. 2025 Oct 9;8:1449. doi: 10.1038/s42003-025-08846-7 (PMC12511343; doi:10.1038/s42003-025-08846-7)
Supplement: Supplementary file 2 — Description of Additional Supplementary Files [file 42003_2025_8846_MOESM2_ESM.pdf]

# Description of Additional Supplementary Files

**File name:** Supplementary Data 1

**Description:** Numerical source data for graphs.

**File name:** Supplementary Movie 1

**Description:** Video showing morph between apo-Cir and MccV-Cir: loop movements

**File name:** Supplementary Movie 2

**Description:** Video showing morph between apo-Cir and MccV-Cir: TonB box movements
