# Supplementary material for: Structural insights into Cir-mediated killing by the antimicrobial protein Microcin V
Source: Commun Biol. 2025 Oct 9;8:1449. doi: 10.1038/s42003-025-08846-7 (PMC12511343; doi:10.1038/s42003-025-08846-7)
Supplement: Supplementary file 6 — Reporting Summary [file 42003_2025_8846_MOESM6_ESM.pdf]

nature portfolio

Corresponding author(s): Susan K. Buchanan
Bryan W. Davies

Last updated by author(s): Aug 26, 2025

Reporting Summary

Nature Portfolio wishes to improve the reproducibility of the work that we publish. This form provides structure for consistency and transparency in reporting. For further information on Nature Portfolio policies, see our Editorial Policies and the Editorial Policy Checklist.

Statistics

For all statistical analyses, confirm that the following items are present in the figure legend, table legend, main text, or Methods section.

n/a	Confirmed	
☐	☒	The exact sample size (*n*) for each experimental group/condition, given as a discrete number and unit of measurement
☐	☒	A statement on whether measurements were taken from distinct samples or whether the same sample was measured repeatedly
☐	☒	The statistical test(s) used AND whether they are one- or two-sided<br>*Only common tests should be described solely by name; describe more complex techniques in the Methods section.*
☒	☐	A description of all covariates tested
☒	☐	A description of any assumptions or corrections, such as tests of normality and adjustment for multiple comparisons
☒	☐	A full description of the statistical parameters including central tendency (e.g. means) or other basic estimates (e.g. regression coefficient) AND variation (e.g. standard deviation) or associated estimates of uncertainty (e.g. confidence intervals)
☒	☐	For null hypothesis testing, the test statistic (e.g. *F*, *t*, *r*) with confidence intervals, effect sizes, degrees of freedom and *P* value noted<br>*Give P values as exact values whenever suitable.*
☒	☐	For Bayesian analysis, information on the choice of priors and Markov chain Monte Carlo settings
☒	☐	For hierarchical and complex designs, identification of the appropriate level for tests and full reporting of outcomes
☒	☐	Estimates of effect sizes (e.g. Cohen's *d*, Pearson's *r*), indicating how they were calculated

Our web collection on statistics for biologists contains articles on many of the points above.

Software and code

Policy information about availability of computer code

Data collection	SerialEM, Gator Plus, Refyn AcquireMP, SA3800 software, BMG Labtech microplate reader software
Data analysis	cryoSPARC, PHENIX, Coot, Isolde, DeepEMhancer, AlphaFold2, QT-Pisa, SEDFIT, SEDNTERP, GUSSI, Gator Plus, Chimera, ChimeraX, Refyn DiscoverMP, FlowJo

For manuscripts utilizing custom algorithms or software that are central to the research but not yet described in published literature, software must be made available to editors and reviewers. We strongly encourage code deposition in a community repository (e.g. GitHub). See the Nature Portfolio guidelines for submitting code & software for further information.

Data

Policy information about availability of data

All manuscripts must include a data availability statement. This statement should provide the following information, where applicable:

- Accession codes, unique identifiers, or web links for publicly available datasets
- A description of any restrictions on data availability
- For clinical datasets or third party data, please ensure that the statement adheres to our policy

The atomic coordinates of the Cir/MccV complex have been deposited at the Protein Data Bank under accession code 9NN6, and the accompanying cryo-EM 3D map has been deposited at the Electron Microscopy Data Bank under accession code EMD-49565. Data supporting these findings are available upon reasonable request. The numerical source data for the graphs in this paper can be found in the provided Supplementary Data file.

Research involving human participants, their data, or biological material

Policy information about studies with human participants or human data. See also policy information about sex, gender (identity/presentation), and sexual orientation and race, ethnicity and racism.

Reporting on sex and gender	N/A
Reporting on race, ethnicity, or other socially relevant groupings	N/A
Population characteristics	N/A
Recruitment	N/A
Ethics oversight	N/A

Note that full information on the approval of the study protocol must also be provided in the manuscript.

Field-specific reporting

Please select the one below that is the best fit for your research. If you are not sure, read the appropriate sections before making your selection.

☒ Life sciences ☐ Behavioural & social sciences ☐ Ecological, evolutionary & environmental sciences

For a reference copy of the document with all sections, see nature.com/documents/nr-reporting-summary-flat.pdf

Life sciences study design

All studies must disclose on these points even when the disclosure is negative.

Sample size	Sample sizes are indicated in figure legends
Data exclusions	CryoEM movies with excessive drift, ice thickness, or poor CTF fit estimation were excluded. Particles lacking apparent TBDT shape/domains were excluded. Channel 6 from representative BLI sensorgram for M33C/V35P Cir mutant omitted due to sensor issue.
Replication	N=3 bactericidal assays and ELISA, N=3 or more with multiple protein preparations for BLI, N=1 for structure determination,
Randomization	N/A
Blinding	N/A

Reporting for specific materials, systems and methods

We require information from authors about some types of materials, experimental systems and methods used in many studies. Here, indicate whether each material, system or method listed is relevant to your study. If you are not sure if a list item applies to your research, read the appropriate section before selecting a response.

Materials & experimental systems

n/a	Involved in the study	
☒	☐	Antibodies
☒	☐	Eukaryotic cell lines
☒	☐	Palaeontology and archaeology
☒	☐	Animals and other organisms
☒	☐	Clinical data
☒	☐	Dual use research of concern
☒	☐	Plants

Methods

n/a	Involved in the study	
☒	☐	ChIP-seq
☐	☒	Flow cytometry
☒	☐	MRI-based neuroimaging

Plants

Seed stocks	N/A
Novel plant genotypes	N/A
Authentication	N/A

Flow Cytometry

Plots

Confirm that:

☒ The axis labels state the marker and fluorochrome used (e.g. CD4-FITC).

☒ The axis scales are clearly visible. Include numbers along axes only for bottom left plot of group (a 'group' is an analysis of identical markers).

☐ All plots are contour plots with outliers or pseudocolor plots.

☐ A numerical value for number of cells or percentage (with statistics) is provided.

Methodology

Sample preparation	E. coli from the Keio collection (BW2113) were grown from single colony isolates in LB to exponential phase. All strains were washed and resuspended in 1xPBS with 50mM glucose.
Instrument	Sony SA3800 spectral cell analyzer
Software	SA3800 software was used for data collection. FlowJo (version 10.10.0) was used for data analysis and visualization
Cell population abundance	10,000 events collected
Gating strategy	Cells were gated using FSC and SSC area. The gating method is described in the methods and an example is shown in Supplementary Figure 10.

☒ Tick this box to confirm that a figure exemplifying the gating strategy is provided in the Supplementary Information.
